# First-year medical students’ calibration bias and accuracy across clinical reasoning activities

**DOI:** 10.1007/s10459-019-09897-2

**Published:** 2019-05-16

**Authors:** Timothy J. Cleary, Abigail Konopasky, Jeffrey S. La Rochelle, Brian E. Neubauer, Steven J. Durning, Anthony R. Artino

**Affiliations:** 1 Graduate School of Applied and Professional Psychology, Rutgers, The State University of New Jersey, New Brunswick, NJ USA; 2 Division of Health Professions Education, Department of Medicine, F. Edward Hébert School of Medicine, Uniformed Services University of the Health Sciences, Bethesda, MD USA; 3 Department of Medical Education, College of Medicine, University of Central Florida, Orlando, FL USA; 4 General Internal Medicine, Walter Reed National Military Medical Center, Bethesda, MD USA; 5 Graduate School of Applied and Professional Psychology, Rutgers, The State University of New Jersey, 152 Frelinghuysen Road, Piscataway, NJ 08854-8085 USA

**Keywords:** Clinical reasoning, Metacognition, Self-assessment, Calibration, Microanalytic assessment, Self-regulated learning

## Abstract

To be safe and effective practitioners and learners, medical professionals must be able to accurately assess their own performance to know when they need additional help. This study explored the metacognitive judgments of 157 first-year medical students; in particular, the study examined students’ self-assessments or calibration as they engaged in a virtual-patient simulation targeting clinical reasoning practices. Examining two key subtasks of a patient encounter, history (Hx) and physical exam (PE), the authors assessed the level of variation in students’ behavioral performance (i.e., effectiveness and efficiency) and judgments of performance (i.e., calibration bias and accuracy) across the two subtasks. Paired *t* tests revealed that the Hx subtask was deemed to be more challenging than the PE subtask when viewed in terms of both actual and perceived performance. In addition to students performing worse on the Hx subtask than PE, they also perceived that they performed less well for Hx. Interestingly, across both subtasks, the majority of participants overestimated their performance (98% of participants for Hx and 95% for PE). Correlation analyses revealed that the participants’ overall level of accuracy in metacognitive judgments was moderately stable across the Hx and PE subtasks. Taken together, findings underscore the importance of assessing medical students’ metacognitive judgments at different points during a clinical encounter.

## Introduction

To be safe, effective, and trustworthy practitioners, medical professionals must continually refine and expand their knowledge, skills, and clinical reasoning abilities (Brydges and Butler 2012; Cruess 2006; Fleet et al. 2008). Moreover, to successfully engage in this form of self-directed learning, individuals need strong metacognitive skills (Cleary et al. 2013), particularly the ability to assess their learning or performance (i.e., self-assessment or calibration). Such skills enable individuals to know when they need to slow down, ask for help, or strive to learn more (Eva and Regehr 2011; Moulton et al. 2007). Yet research indicates that medical practitioners are not very accurate in this self-assessment or calibration; that is, there is misalignment of their own metacognitive judgments of performance with other internal or external standard (Blanch-Hartigan 2011; Bol and Hacker 2012; Chen and Rossi 2012; Davis et al. 2006; Epstein 2007; Eva and Regehr 2011; Norman et al. 2004; Pieschl 2009).

Poor calibration among medical professionals has led to concerns regarding patient safety, especially when considering less-skilled practitioners who are more likely to overestimate their competencies and skill sets (Blanch-Hartigan 2011; Davis et al. 2006; Ehrlinger et al. 2008; Kruger and Dunning 1999). Recent work, however, suggests that poor calibration may be, in part, a function of the granularity of measurement and the complexity of a given clinical activity (Blanch-Hartigan 2011; Bol and Hacker 2012; Davis et al. 2006; Eva and Regehr 2007, 2011). To explore this issue, the current study examined variations in the calibration skills of novice practitioners (i.e., medical students) across different subtasks of a clinical reasoning activity.

### Granularity of calibration: the importance of sub-tasks

Much of the self-assessment or calibration literature, particularly in health professions education, is grounded in a methodology that focuses on *overall* performance on a task or activity—such as test score, Objective Structure Clinical Exam (OSCE) performance, or class grade—rather than on *specific* elements of that task (Blanch-Hartigan 2011; Bol and Hacker 2012; Davis et al. 2006). In response to this lack of granularity, Eva and Regehr (2005, 2007, 2011) undertook a line of inquiry to understand the components of self-assessment or calibration and ways to improve it. They describe self-assessment in terms of a process, ranging from prediction (i.e., before performance) to assessment and adjustment (i.e., during performance) to post-diction (i.e., judgments after performance). Through these investigations of process, they determined that the accuracy of metacognitive judgment depends in part on its *granularity*. For instance, self-assessment of one’s overall skill in a clinical area tends to be less accurate than evaluative performance judgments for a specific clinical task. Self-assessment also tends to be less accurate in open-ended clinical encounters (with fewer clearly delineated subtasks) relative to more narrow, written assessments (Eva and Regehr 2005, 2007, 2011). Thus, the broader and more ambiguous an activity, the greater the likelihood that individuals will make less accurate judgments of their performance.

In response to similar trends in other educational contexts, educational psychologists have begun to assess calibration judgments in a more fine-grained manner. That is, calibration has been assessed across distinct subtasks for a given learning activity or across several types of questions on a particular test (Bol and Hacker 2012; Chen 2003; Dunlosky and Hertzog 2000; Pieschl 2009; Pressley and Ghatala 1988). For example, Pressley and Ghatala examined the accuracy of students’ performance estimates across different types of questions within a broader task and found differences in calibration accuracy (i.e., less accuracy for more complex or ambiguous question types; Pressley and Ghatala 1988). Medical education researchers are also beginning to recognize the relevance of examining calibration at different parts of a clinical encounter (e.g., at different points during a physical exam or interview; Eva and Regehr 2007, 2011), in part, because many common clinical activities are multi-faceted and complex. To our knowledge, however, there is limited research examining the variability in metacognitive judgments across *subtasks* for specific clinical activities, with even fewer studies addressing this issue when medical students engage in clinical reasoning during a patient encounter. In order to better help clinicians identify knowledge gaps and instructional needs, it is important for researchers to conduct more fine-grained analyses of individuals’ metacognitive or regulatory processes and to explore potential shifts or changes in these processes during a particular clinical activity (Cleary et al. 2015; Eva and Regehr 2011; Pieschl 2009; Sargeant et al. 2010).

### Calibration of clinical reasoning and avoiding diagnostic error

Enhancing the granularity of measurement in medical education is particularly important given the growing body of research in clinical reasoning and the consequences of diagnostic error (National Academies of Sciences and Medicine 2016; Singh and Graber 2015). Diagnostic errors are now cited as the third leading cause of death behind heart disease and cancer (Makary and Daniel 2016). In fact, a recent study suggests that mis-calibration is a key contributing factor to these errors; when study participants (i.e., general internists) overestimated their performance, they were less likely to seek additional diagnostic tests which, unfortunately, may have led to higher error rates (Meyer et al. 2013). Clinical reasoning involves a series of distinct, albeit interconnected, activities—some conscious and analytical, some more intuitive and non-analytical (e.g. pattern recognition, Norman et al. 2017)—needed to arrive at a correct diagnosis and a case-specific management plan (Cook et al. 2018; Young et al. 2018). During a clinical encounter, a medical professional will typically take a patient history (Hx), perform a physical exam (PE), and possibly run a series of diagnostic tests, all while concurrently synthesizing and integrating information to identify the most likely diagnosis. Most importantly, clinicians must *actively adjust* their approach in line with an external criterion (i.e., a case-specific diagnosis and treatment). Given this complexity, clinical reasoning can be difficult to define, assess, and study.

In order to optimally understand these moment-by-moment adjustments and offer interventions to improve them, researchers must examine metacognitive judgments and use of strategies at a sufficient level of granularity (Pieschl 2009). This entails conceptualizing assessment “as a multifaceted construct comprising numerous discrete activities” (Sargeant et al. 2010, p. 1212), including data collection, self-awareness, interpretation, critical reflection, and professional climate (and including environmental and relationship tensions). Such an approach suggests that there is value in de-aggregating the holistic, integrated process of clinical reasoning into specific subtasks and examining the nature and pattern of individuals’ thought processes and/or actions across these subtasks. In this way, calibration accuracy might improve, perhaps redeeming self-assessment as a valuable (and predictive) clinical skill.

### Purpose of the current study

In this study, we focus on the clinical subtasks of history (Hx) and physical exam (PE) because they are commonly taught as distinct elements in medical education (cf., the widely used *Bates’ Guide to Physical Examination and History Taking*, Bickley and Szilagyi 2012). Further, although Hx and PE work in concert to facilitate the identification of the diagnosis, the Hx component may be more complex and ambiguous as it often focuses more on *diagnostic generation* (e.g., questioning to address an oftentimes vague chief complaint) while the PE typically tends to be more about *diagnostic confirmation* (e.g., physical palpation or visual inspection to confirm something raised in the history). Thus, clinicians will often have more refined hypotheses by the time they reach the PE since it follows the history. Further, Fischer and Budescu (2005) argue that *screening for* potentially relevant categories (as in the Hx) is more difficult to calibrate than *discrimination among* identified categories (as in the PE; Fischer and Budescu 2005).

Two common metrics for examining calibration are *bias* and *accuracy*. Calibration bias represents the valence (or direction) of performance judgment errors that learners make (i.e., overestimation or underestimation), while calibration accuracy reflects the magnitude of those errors (Bol and Hacker 2012; Pajares and Graham 1999; Pieschl 2009). In the current study, we examined variations in the metacognitive judgments (i.e., calibration accuracy and calibration bias) of medical students across Hx and PE subtasks of a clinical reasoning activity embedded in a virtual-patient simulation. We asked three research questions: (1) Are there differences in medical students’ observed performance (i.e., effectiveness and efficiency) across Hx and PE subtasks?; (2) Are there differences in medical students’ metacognitive performance judgments (i.e., calibration accuracy and bias) across Hx and PE?; and (3) Are medical students’ metacognitive judgments stable across Hx and PE? For the first two research questions, we hypothesized that Hx-PE differences in performance, calibration bias, and calibration accuracy would emerge. Specifically, given that Hx is typically a conceptually more complex and ambiguous task than PE, we hypothesized that students would perform better and display more adaptive metacognitive judgments on the PE subtask than Hx. For the third research question, we did not specify an *a priori* hypothesis given the exploratory nature of question.

## Method

The present study was conducted at the F. Edward Hébert School of Medicine, Uniformed Services University of the Health Sciences (USU), Bethesda, MD, USA during the 2013–2014 academic year. The study was approved by the university’s Institutional Review Board.

At the time of the study, USU offered an integrated, organ-system-based curriculum comprised of the following three phases: (1) an 18-month pre-clerkship phase that included five distinct modules integrating basic science knowledge and clinical skills in an organ-system based approach; (2) a 12-month core clerkship phase; and (3) an 18-month post-clerkship phase that included a six-week period of advanced didactics and a 12-month period of advanced clinical rotations (i.e., clinical electives, a military field exercise, and an opportunity to complete a scholarly capstone project).

### Participants and educational context

A sample of first-year medical students was invited to participate in the study. The students were recruited from an Introduction to Clinical Reasoning (ICR) course. Volunteers were offered extra credit points for participating, while non-participants could earn the same extra credit points through an alternate means (i.e., a short, reflective writing assignment). Prior to the ICR course, the students had not received any formal didactic or clinical experience in clinical reasoning. Therefore, for the purpose of this study, the participants were considered novice learners. The course itself was comprised of a series of large- and small-group activities. Each course component was designed to expose students to various symptoms, PE findings, laboratory test abnormalities, and syndromes.

### Procedures

All participants were asked to complete the same clinical case using a virtual-patient simulation system called i-Human Patients (i-Human Patients, Inc., Sunnyvale, California). i-Human simulates a complete patient encounter to include taking a history, performing a PE, ordering tests, and making an appropriate differential diagnosis and management plan.

Students were asked to engage with a virtual case across the following sections: Hx, PE, assessment (e.g., problem list, hypothesis selection), and management plan. For the purposes of this study, however, we focused specifically on the Hx and PE sections. The chief complaint for the virtual patient was *progressive fatigue with shortness of breath*, and the leading diagnosis was *iron*-*deficiency anemia*. The virtual case was developed by two of the authors who are experienced clinicians (SD and JL). Evidence of content validity for the case was collected by obtaining qualitative reviews by four additional expert clinicians. During the simulated clinical encounter, the i-Human system was programmed to administer questions addressing metacognitive judgments of performance for both Hx and PE sections. Participants were also asked to provide a differential diagnosis after completing the Hx and PE. There was no time limit for completing the case.

### Measures

Students’ *observed performance* and *metacognitive judgments of performance* were assessed across both the Hx and PE subtasks of the patient encounter.

#### Patient history

Student performance on Hx was assessed in two ways: effectiveness and efficiency. *Hx effectiveness* was defined as the percentage of essential (i.e., expert-endorsed) questions asked by the learner (selected via a drop-down menu) relative to the total number of expert-endorsed questions for this case. For instance, an effectiveness score of 60% indicates that of all the expert-endorsed questions that should be asked during the Hx, the learner asked 60% of those questions. Conversely, *Hx efficiency* was defined as the percentage of essential questions asked relative to the total number of questions (essential and non-essential) asked by the learner. Thus, an efficiency score of 25% indicates that one quarter of all questions asked by the learner reflected expert-endorsed questions. To identify the expert-endorsed or essential questions, two internal medicine physicians (SD and JL) reviewed all questions included in the drop-down menu for this case. These questions were then reviewed by four other experts in internal medicine, revised, and then re-discussed until full consensus was reached.

After students completed the Hx section, they were asked the following post-diction question, “Approximately what percentage of the questions that you asked would an expert clinician say were essential questions for this case? (Indicate a percentage between 0 and 100%.)” Two dimensions of metacognitive judgments were calculated: *calibration bias* and *calibration accuracy* scores. Consistent with previous research (Cleary and Chen 2009; Pajares and Graham 1999), calibration bias was calculated as the *difference* between actual performance (i.e., using efficiency score) and a judgment of performance (i.e. post-diction). Calibration bias scores ranged from − 100 to + 100 with positive values indicating overestimation of performance and negative values indicating underestimation of performance. As an example, if a learner estimated that 95% of the questions that she asked during Hx were essential to the case (post-diction) but only 40% were actually endorsed by the experts, then she would exhibit a positive calibration bias score of + 55 (i.e. 95 − 40). Such a score would be an indicator of overestimation (with 0 being perfect calibration).

*Calibration accuracy* represented the *magnitude* of the judgment error and was calculated by subtracting the absolute value of the Hx bias score from the maximum value on the performance scale (i.e. 100; Pajares and Graham 1999). For example, if the learner received a bias score of + 55 (as in the previous example), her accuracy score would be 45 (i.e., 100 − 55). If a different participant had a bias score of − 20, which represents a level of underestimation of her ability to conduct a Hx, her accuracy score would be 80 (100 − 20). An accuracy score of 100 denotes perfect accuracy (no error in judgment) whereas a score of 0 represents complete misjudgment.

#### Physical exam

Effectiveness and efficiency scores were also calculated for the physical exam component. *PE effectiveness* was defined as the percentage of essential exams administered by the learner (selected via a drop-down menu) relative to the total number of exams identified by experts as essential for this case. Conversely, *PE efficiency* was defined as the percentage of expert-endorsed exams administered by the learner relative to the total number of potential exams (essential and non-essential) administered by the learner. After students completed the PE, they were asked to respond to the following judgment of performance question, “Now that you have completed the physical examination, approximately what percentage of the exams that you conducted would an expert clinician say were essential in this case? (Indicate a percentage between 0 and 100%).” Calibration bias and calibration accuracy scores for PE were calculated using procedures identical to those for Hx.

### Analysis

After screening the data for missing values, we conducted paired *t*-tests (one-tailed) to examine differences in performance and performance estimates across the Hx and PE components of the patient encounter. Next, we conducted correlation analyses to assess the stability of bias and accuracy estimates across Hx and PE subtasks.

## Results

Of the 172 first-year medical students invited to participate, 168 (98%) completed the study. However, 11 individuals did not answer the metacognitive judgment question for either the Hx or PH subtask and thus were removed prior to conducting any statistical analyses. The final sample used for analysis was 157, which included 102 men (65%) and 55 (35%) women. This gender breakdown is similar to the overall medical student population at USU at the time of the study (71% men).

### Within-group differences across Hx and PE

Table 1 presents the descriptive statistics for the primary variables. Based on paired *t* test analyses, the participants showed weaker performance on the Hx portion of the clinical encounter than PE, as evidenced by lower effectiveness and efficiency scores. In terms of effectiveness, the students used, on average, a significantly lower percentage of essential (i.e., expert-endorsed) Hx questions (*M* = 43.07%) than essential exam actions during PE (*M* = 71.63%): *t* (156) = 19.85, *p* < .05. This observed difference is considered very large (Cohen’s *d* = 1.57). A similar pattern emerged for efficiency (i.e., essential questions or exams/total questions or exams). The results revealed that only 17% of all Hx questions asked by the learners were considered essential Hx questions, whereas 34% of all exam actions conducted by learners were identified as essential. Thus, the students were less efficient in their use of Hx questions than in the PE actions. This difference was statistically significant and considered extremely large: *t* (156) = 19.05, *p* < .05, *d* = 2.21.

**Table 1:** Summary of descriptive statistics for Hx and PE performance, post-diction, and calibration scores

Clinical task	Effectiveness M % (SD)	Efficiency M % (SD)	Post-diction M % (SD)	Bias M % (SD)	Accuracy M % (SD)
Patient history (Hx)	43.07 (17.36)	17.24 (7.03)	63.23 (21.17)	45.99 (20.43)	53.95 (20.29)
Physical exam (PE)	71.63 (17.06)	34.19 (11.78)	69.83 (20.14)	35.64 (20.53)	62.72 (17.71)
Difference (PE-Hx)	− 28.56* (18.03)	− 16.95* (11.15)	− 6.60* (19.71)	10.35* (22.01)	− 8.78* (20.76)
Cohen’s *d*	1.57	2.21	.33	.48	.40

Positive bias scores indicate overestimation. High accuracy scores indicate greater levels of accuracy

*p < .05

Hx-PE differences in metacognitive judgments were also examined. Almost all participants overestimated their performance to some degree on both Hx (*n* = 154; 98%) and PE (*n* = 149; 95%), but participants exhibited significantly higher performance estimates for PE (*M* = 69.83%) than Hx (*M* = 63.23%), *t* (156) = 4.19, *p* < .05. That is, they perceived that they performed better on the PE subtask than the Hx subtask. This difference is considered small to medium (Cohen’s *d* = .33).

Finally, we calculated two types of calibration scores (i.e., bias and accuracy) to generate a more nuanced understanding of the participants’ performance judgments. For bias, we observed that although the learners overestimated (i.e. positive calibration bias) their skills on both Hx (*M* = + 45.99%) and PE (*M* = + 35.64%), they showed a greater level of overestimation for Hx, *t* (156) = 5.89, *p* < .05. The magnitude of this difference is considered medium (Cohen’s *d* = .48).

In terms of calibration accuracy, scores of 100 indicate perfect accuracy whereas scores of 0 represent complete inaccuracy. We found that although participants showed inaccuracy across both Hx (*M* = 53.95%) and PE (*M* = 62.72%), they showed significantly lower accuracy scores for Hx, *t* (156) = 5.30, *p* < .05. This observed difference is considered medium (Cohen’s *d* = .40).

### Relations among calibration scores

Pearson and point-biserial correlations were calculated to examine the relations among calibration scores (see Table 2). We were specifically interested in the correlations between the Hx and PE bias scores and between the Hx and PE accuracy scores. To facilitate accurate interpretation of the bias scores (over-estimation or under-estimation), which were substantially range restricted, we elected to create a dichotomous variable (i.e., underestimation = 0, overestimation = 1). The results revealed a small, positive association between Hx and PE bias scores (*r* = .18, *p* < .05) and medium, positive relation between accuracy scores (*r* = .41, *p* < .05). Thus, participants who overestimated their performance on Hx were more likely to overestimate on PE. Similarly, students who exhibited high levels of inaccuracy on Hx were more likely to report high levels of inaccuracy on PE.

**Table 2:** Correlations among calibration bias and accuracy scores

	Bias: history	Bias: physical exam	Accuracy: history
Bias: history	–		
Bias: physical exam	.18*	–	
Accuracy: history	− .31*	− .22*	–
Accuracy: physical exam	.08	− .28*	.41*

Bias scores were based on a dichotomous dummy variable: 0 (under-estimators), 1 (over-estimators)

Accuracy scores were based on a scale ranging from 0 to 100, with higher scores indicating greater levels of accuracy

*p < .05

## Discussion

The present study examined differences in first-year medical students’ metacognitive judgments across two subtasks (i.e., Hx and PE) of a clinical reasoning activity embedded within a virtual-patient case. This study is important because it underscores the premise that students’ performance and the quality of their metacognitive processes will often vary across different parts or components of a single clinical activity. We believe these findings have important implications for assessment and feedback practices in medical education, particularly when students are asked to perform difficult and comprehensive clinical activities; two task features that can negatively affect calibration (Lin and Zabrucky 1998).

### Differences between Hx and PE

As hypothesized, the primary finding in this study was that students’ performance and metacognitive processes showed significant variation across the Hx and PE subtasks of a virtual-patient encounter. As reflected in Table 1, these differences were observed across all performance (i.e., effectiveness, efficiency) and metacognitive measures (i.e., bias and accuracy in post-diction). Thus, for the particular case employed in our study (i.e., iron deficiency anemia), the Hx subtask was found to be a more challenging and complex activity than PE. In terms of performance, the participants used fewer of the “ideal” or expert-endorsed Hx questions and were significantly less efficient in their use of questions during Hx, relative to their use and efficiency in exam actions during the PE subtask. In addition, participants showed significantly higher levels of overestimation and overall inaccuracy in their judgments of performance on Hx than on PE.

Although we did not explore the *causes* of these metacognitive judgment differences, our results appear to convey that Hx is indeed a more complex task and/or may involve a greater number of associated parts; features which may make it more challenging to accurately assess performance on this subtask relative to PE (Lin and Zabrucky 1998; Pieschl 2009). The Hx subtask occurred at the beginning of the clinical encounter and thus necessitated students to select from a large number of potential questions to address a multitude of conditions or presenting symptoms. In contrast, PE activities occur towards the end of the encounter and tend to be more fixed in terms of degrees of freedom and how physical findings relate to one another. In other words, the PE task is more about discriminating among a smaller set of targets (Fischer and Budescu 2005), which likely cuts down on the ambiguity and overall demands experienced during Hx (Lin and Zabrucky 1998).

In terms of the nature of the participants’ miscalibration, descriptive analysis revealed that almost all individuals overestimated their performance on both Hx (*n* = 154; 98%) and PE (*n* = 149; 95%). Thus, the average participant believed that he performed better than he had actually performed. While overestimation is a common pitfall for novices performing complex tasks, it is noteworthy that we measured metacognitive judgments via post-dictions (i.e., metacognitive judgments made *following* a subtask) rather than predictions (i.e., metacognitive judgments made *prior* to a subtask). Typically, post-dictions should be more accurate than predictions because participants get access to information about the nature and demands of a given activity before they are asked to make a metacognitive judgment (Pieschl 2009). Our results are noteworthy because such a large percentage of individuals overestimated their performance on both subtasks even though they had just completed the activities. Overestimation is highly problematic because it can lead individuals to believe that they do not need to adapt or improve when they are in fact struggling or underperforming on an activity (Blanch-Hartigan 2011; Chen and Bembenutty 2018).

As mentioned previously, clinical reasoning is often recognized to be a holistic process that is impacted both by content (content specificity) and the specifics of the situation (context specificity, Durning et al. 2012); yet, medical educators also recognize the value of categorizing the subtasks of clinical reasoning, such as assessing priorities and determining and refining diagnosis and treatment plans (Juma and Goldszmidt 2017). As illustrated in this study, breaking clinical reasoning into subtasks may help medical educators become better equipped to identify knowledge gaps in the skills of trainees, particularly given that inexperienced or novice clinicians are often largely unaware of these gaps (Blanch-Hartigan 2011; Davis et al. 2006; Kruger and Dunning 1999). Moreover, examination of subtasks or components of a clinical activity may help to identify medical students or clinicians who may be juggling multiple reasoning tasks and, hence, experiencing high cognitive load or high mental effort (Juma and Goldszmidt 2017). The ability to identify such struggling individuals is both timely and emergent given the current crisis of medical errors exhibited by physicians in this country (Makary and Daniel 2016).

### Relations among calibration measures and performance

Whereas the first two research questions examined differences in performance and metacognitive judgments across the two clinical reasoning subtasks, our third research question investigated the extent to which the nature of calibration bias (i.e., overestimation, underestimation) and accuracy judgments among individuals would show stability across subtasks. Although we observed statistically significant relations between Hx and PE bias scores (*r* = .18) and between Hx and PE accuracy scores (*r* = .41), the effect size was larger for accuracy scores. Thus, students who displayed high levels of inaccuracy on Hx were moderately more likely to show high levels of inaccuracy on PE. In terms of the small correlations observed between the bias scores, these results may have been due, in part, to a restriction of range across the two bias categories (i.e., over- and under-estimation). In fact, we only observed 3 under-estimators (i.e., 2%) for Hx bias and 8 under-estimators (5%) for PE bias. Thus, although the finding that the large majority of participants overestimated their performance was consistent with prior research (Blanch-Hartigan 2011; Davis et al. 2006), the restriction of range may have adversely affected the size of the observed relation.

When these findings are viewed in conjunction with the results from the first two research questions, it appears that there is a tendency for novice learners to exhibit similar patterns of inaccuracy across clinical subtasks, but that the *level* of that inaccuracy will vary as a function of the subtask. Thus, although the accuracy of the learners’ metacognitive judgments may be somewhat stable across subtasks, students may experience varying levels of success across those subtasks. The primary implication, which is discussed in greater detail in the implication section, is that assessment approaches that can generate information about students’ varying level of performance and cognitive processes across different aspects of complex clinical tasks may enable both medical educators and their students to more fully understand the underlying sources of potential struggles.

### Limitations

There are a few important limitations in this study. First, we utilized a sample of medical students from a single institution and focused on only one clinical scenario in a simulated, virtual-patient environment that lacked the authenticity of an actual clinical encounter. Therefore, the external validity of the results is quite limited. This limited external validity is especially important if one considers the well-known challenges associated with case specificity (ten Cate and Durning 2018; Wimmers and Fung 2008). Therefore, it is important to replicate this study across contexts, content, samples, and clinical tasks to corroborate the consistency of the results. Although Hx and PE are two critical activities for arriving at a correct diagnosis (Bickley and Szilagyi 2012; National Academies of Sciences and Medicine 2016), our study was also limited because it only targeted these two activities. Future research can expand the design and scope of the current study by examining calibration bias and accuracy judgments across other components of the clinical process (e.g., ordering tests), and also by gathering data about the accuracy of the diagnoses generated at different points during this process or perhaps even breaking Hx down into further component parts.

It is also noteworthy that the overall scope of metacognition and self-regulation targeted in this study was fairly narrow. Concurrently examining metacognitive judgments along with other regulatory (e.g., strategy use, planning, self-evaluation) and motivational (e.g., self-efficacy) processes can be useful for more fully understanding differences across subtasks of clinical activities.

### Implications and future directions

Our results support recent research in medical education indicating that while clinical reasoning can be conceptualized as a holistic process, there is value in also viewing it as a series of subtasks (Juma and Goldszmidt 2017). Further, it is important for medical educators and researchers to recognize that a range of skills is typically needed to succeed on most clinical activities, such as identifying symptoms, considering contextual factors, integrating data, and comparing and contrasting diagnoses during a patient encounter, and that novice learners may exhibit a distinct profile of skills, beliefs, and behaviors across different parts or situations of such activities (Sargeant et al. 2010). Thus, medical education researchers should not only seek to understand how clinicians’ performance differs across subtasks in clinical reasoning, but also the quality with which they *think about* and *evaluate* that performance.

While a recent review and commentary point to major gaps in self-regulation research in clinical contexts (Gandomkar et al. 2018; van Houten-Schat et al. 2018), there have been advances in assessment methodologies used in medical education research (Andrews et al. 2016; Artino et al. 2014; Cleary et al. 2015). These methodologies have enabled researchers and educators to conduct more nuanced and contextualized assessments of clinicians’ cognitive, behavioral, and metacognitive processes as they engage in clinical activities. For example, Cleary, Dong, and Artino used a microanalytic assessment protocol to examine shifts in the motivational beliefs and regulatory processes of medical students during a virtual-patient encounter (Cleary et al. 2015). This methodology enabled the authors to identify statistically significant shifts in the students’ self-efficacy and regulatory processes in response to negative feedback regarding the accuracy of their differential diagnosis during a clinical encounter. We believe that using microanalytic protocols, think alouds, or other types of fine-grained assessments (i.e., calibration assessment) can yield valuable information about regulatory processes in general. Further, in addition to administering calibration assessments across different subtasks (Hx, PE; as was the case in the current study) of a given clinical activity, it would be useful for researchers to administer such tools at* multiple points* during a given subtask rather than wait until after a task is completed to gather a more aggregate judgment (as was the case in the current study). In doing so, such an approach can also help educators provide more nuanced and contextualized feedback to learners as they work through any complex clinical activity, including clinical cases that are incorporated into high-stakes assessments (Andrews et al. 2016).

This focus on enhancing the nuanced nature and granularity of measurement in medical education is particularly important considering the crisis of diagnostic medical errors (Makary and Daniel 2016). In fact, a recent study suggests that mis-calibration is a key contributing factor to these errors; when study participants (general internists) overestimated their performance, they were less likely to seek additional diagnostic tests which, unfortunately, may have led to a higher error rate (Meyer et al. 2013). In order to address mis-calibration, medical educators and researchers should focus on two critical initiatives: (a) clearly demarcating the underlying subtasks of core clinical reasoning activities performed by medical students or clinicians, and (b) using assessment approaches that target the behavioral, cognitive, and/or metacognitive processes during the activities. If medical educators do not strategically focus on the nature of the tasks or activities that they expect students or clinicians to perform, they will find it quite challenging to identify and provide data-informed remedial or intervention supports to optimize learning. This is an especially important initiative for trainees, given that many of these learners exhibit skill deficits and typically lack adequate self-awareness to self-correct and improve on their own (Kruger and Dunning 1999). Thus, future research needs to carefully consider how to strategically “break up” clinical tasks so that performance and regulatory processes can be meaningfully assessed. To this end, researchers should collaborate with experts in a specific clinical activity to identify the most relevant and important subparts and then to structure the assessment protocols around these component parts.

Finally, the current study suggests that health professional contexts can offer valuable insight into the broader discussion of calibration and metacognition in psychology and education. The sheer number of potential subtasks and complex processes involved in clinical reasoning (Juma and Goldszmidt 2017) offers a vibrant testing ground for theories about the component parts of calibration and the metacognitive strategies trainees and professionals use to actively adapt to emerging task complexity (Pieschl 2009).
